# Between-Trial Forgetting Due to Interference and Time in Motor Adaptation

**DOI:** 10.1371/journal.pone.0142963

**Published:** 2015-11-24

**Authors:** Sungshin Kim, Youngmin Oh, Nicolas Schweighofer

**Affiliations:** 1 Neuroscience Graduate Program, University of Southern California, Los Angeles, 90089, United States of America; 2 Biokinesiology and Physical Therapy, University of Southern California, Los Angeles, 90089, United States of America; 3 M2H Laboratory, Euromov, University of Montpellier I, Montpellier, France; VU University Amsterdam, NETHERLANDS

## Abstract

Learning a motor task with temporally spaced presentations or with other tasks intermixed between presentations reduces performance during training, but can enhance retention post training. These two effects are known as the spacing and contextual interference effect, respectively. Here, we aimed at testing a unifying hypothesis of the spacing and contextual interference effects in visuomotor adaptation, according to which forgetting between trials due to either spaced presentations or interference by another task will promote between-trial forgetting, which will depress performance during acquisition, but will promote retention. We first performed an experiment with three visuomotor adaptation conditions: a short inter-trial-interval (ITI) condition (SHORT-ITI); a long ITI condition (LONG-ITI); and an alternating condition with two alternated opposite tasks (ALT), with the same single-task ITI as in LONG-ITI. In the SHORT-ITI condition, there was fastest increase in performance during training and largest immediate forgetting in the retention tests. In contrast, in the ALT condition, there was slowest increase in performance during training and little immediate forgetting in the retention tests. Compared to these two conditions, in the LONG-ITI, we found intermediate increase in performance during training and intermediate immediate forgetting. To account for these results, we fitted to the data six possible adaptation models with one or two time scales, and with interference in the fast, or in the slow, or in both time scales. Model comparison confirmed that two time scales and some degree of interferences in either time scale are needed to account for our experimental results. In summary, our results suggest that retention following adaptation is modulated by the degree of between-trial forgetting, which is due to time-based decay in single adaptation task and interferences in multiple adaptation tasks.

## Introduction

It has been known for more than a century that manipulating the schedules of motor training affects performance during training and retention [1–3]. In particular, practicing a task with temporally spaced presentations or with other tasks intermixed between presentations reduces performance during training compared to blocked presentations, but can lead to superior retention. These phenomena are known as the spacing effect and the contextual interference effect, respectively [4–7].

According to the “forgetting-reconstruction” theory of the contextual interference effect, short-term forgetting between presentations of the same task results in stronger memories [8]. Such a forgetting view had previously been advanced to explain the spacing effect in verbal learning [9]. Lee and Magill then proposed a unifying mechanism of the spacing and contextual interference effects: both spacing between presentations and interference by another task promote forgetting in working memory, which will depress performance during acquisition, but will promote retention [5].

However, to the best of our knowledge, no study has directly compared spaced and intermixed practice in motor adaptation. Our goal here was therefore two-fold. First, we aimed at directly testing the effects of temporally spaced presentations and task-intermixed presentations in visuomotor adaptation. Specifically, we compared performance during and after training in three visuomotor adaptation conditions: a short inter-trial-interval (ITI) condition with a single task (SHORT-ITI); a long ITI condition with a single task (LONG-ITI); and an alternating condition with two alternated opposite tasks (ALT), with the same single-task ITI as in LONG-ITI. In this design, the only difference between LONG-ITI and ALT schedules was the intercalation of a secondary task in ALT. We could therefore estimate the effect of forgetting due to time by comparing LONG-ITI to SHORT-ITI, and the effect of forgetting due to interference by comparing LONG-ITI to ALT.

Second, using a combined approach of computational modeling and behavioral experiment, we aimed at testing the unifying mechanism of the spacing and contextual interference effects akin to that proposed by Lee and Magill but for visuomotor adaptation. One important advantage of computational models is the ability to estimate latent variables underlying adaptation and to make predictions based on the estimated variables [10–13]. In particular, in the two-state model [12], a fast learning process contributes to fast initial learning but forgets quickly, and a slow learning process contributes to long-term retention but learns slowly. It has been proposed that rapid forgetting in the fast process can produce the spacing effect [14]: in conditions with short ITIs, there is little forgetting between consecutive presentations of the task, and performance improves quickly because of large update of the fast process. In contrast, in conditions with long ITIs, there is significant fast process forgetting between trials: performance improves slowly, and the larger errors result in greater updates of the slow process, leading to increased long-term retention.

The two-state model cannot, however, explain data on multiple task adaptation and thus the effect of interfering tasks, because adaptation to a new task overrides previous adaptation in this model. When given appropriate contextual cues, humans can simultaneously adapt to two visuomotor rotations [15–17], two saccadic gains [18], and even, in some cases, two opposite force fields [19, 20]. To account for such data, we previously proposed an updated model with a “common fast process”, which is highly prone to interference and competes for errors, and with multiple slow processes protected from interference [21]. In simulations of this model in an alternating schedule with two adaptation tasks of opposite signs, the fast process was interfered by the second alternating adaptation task. This interference induced trial-by-trial forgetting during training and thus reduced overall adaptation rate, thereby increased errors leading to greater update of the slow process, and thus increased retention as in the contextual interference effect [21]

We analyzed patterns of visuomotor adaptation and forgetting from the three experimental conditions, SHORT-ITI, LONG-ITI, and ALT. Guided by the “forgetting-reconstruction” theory, and based on our previous simulations [22], we then proposed to test the “between-trial forgetting, long-term retention hypothesis” (BTF-LTR), which makes the following predictions. The amount of between-trial forgetting during training is predicted to be smallest in the SHORT-ITI, largest in the ALT, and intermediate in the LONG-ITI. Therefore, in the SHORT-ITI, we predicted fastest increase in performance during training and largest short-term forgetting in the retention tests. In contrast, in the ALT, we predicted slowest increase in performance during training and smallest short-term forgetting in the retention tests. Compared to these two conditions, in the LONG-ITI, we predicted intermediate increase in performance during training and short-term forgetting. We then studied what models of adaptation can account for our results by fitting and comparing six possible adaptation models with one or two time scales, and with interference in the fast time scale, the slow time scale, or both time scales.

## Methods

### Participants

Forty-six neurologically intact right-handed subjects (10 men and 36 women, 21–32 years old) participated in the study. We randomly assigned the subjects to one of three different experimental conditions: SHORT-ITI, LONG-ITI, and ALT, with a predefined goal of 15 subjects per group. Participants were excluded from the study if the standard deviation of directional error between the target and the final cursor position in the first 80 trials of the familiarization session was greater than 10 degrees (see below). One participant was excluded according to this criterion. The subjects, who were naïve to the purpose of the study, signed an informed consent form prior to participation in this study, which was approved by the Institutional Review Board at the University of Southern California.

### Experimental procedures

**Fig 1:**
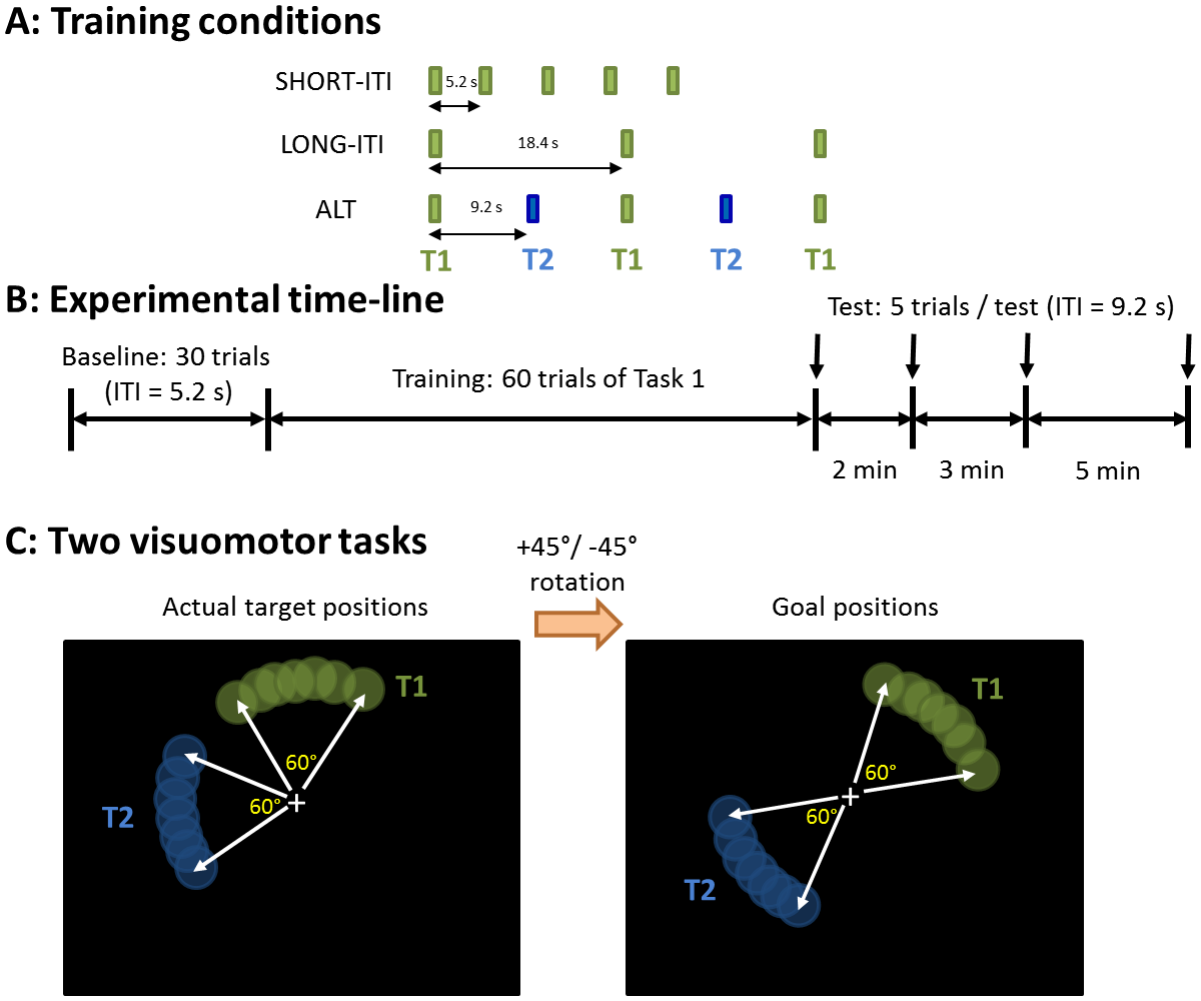
Experiment design. ***A***: Training schedules of the three visuomotor adaptation experimental groups. Note that the schedule for each task in ALT is the same as the single task schedule in LONG-ITI. The only difference between ALT and LONG-ITI is the second task (T2) is intercalated between two presentations of the first task (T1). ***B***: The experiment consisted of three blocks: 30 baseline trials, 60 training trials per task (which depend on the condition, as shown in ***A***), and retention test trials, given in blocks of 5 trials at 2 min, 5 min, and 10 min after the end of training trials. ***C***: Distribution of targets: Targets randomly appeared either upward or leftward depending on a task with a color cue, green or blue. To hit a target, subjects had to adapt to the altered the mapping between the hand position and the cursor position via either a counterclockwise (CCW: +45°) visuomotor rotation or a clockwise (CW: -45°) rotation depending on a task.

Design: Subjects sat facing a computer monitor with the right arm supported with a JAECO/Rancho arm support. They controlled a cursor shown on a screen by moving a pen on the surface of a digitizing tablet (sampling rate: 200 Hz, Wacom Tech Corp). An opaque shield blocked vision of their hand and arm. At each trial, a target appeared and subjects were instructed to make a straight and uncorrected out-and-back movement to hit a target. The ITI was defined as the time interval between onsets of two consecutive targets. An initial familiarization session (140 trials with full feedback, 20 trials without feedback) was followed by a block of 30 baseline trials. In both familiarization and baseline, the mapping between the hand position and the cursor was unaltered, and ITI was 5.2 s. In the following training trials, we altered the mapping between the hand position and the cursor position via either a counterclockwise (CCW: +45°) or a clockwise (CW: -45°) visuomotor rotation. The schedules of the training trials varied according to the experimental conditions (Fig 1*A*). In SHORT-ITI and LONG-ITI conditions, 60 trials of either a CCW or a CW rotation (counterbalanced across subjects) were presented, with ITIs of 5.2 s and 18.4 s, respectively. In ALT conditions, both CCW and CW rotations were presented alternatively with an ITI of 9.2 s, with 60 trials per task (120 trials in total). Thus, the ITI for each task in ALT was 18.4 s, which is the same as the ITI for the LONG-ITI condition.

Then, four blocks of retention tests, each consisting of five trials (ITI = 9.2 s) without feedback, were given at 0 min, 2 min, 5 min, and 10 min after the end of training (Fig 1*B*). For SHORT-ITI and LONG-ITI, the practiced task (CCW or CW) was presented in the retention tests. For ALT, the first practiced task was presented in the retention tests. The ITI following the last training trial before the retention trials was 5.2 s for all three groups to measure immediate retention.

Single trial: At the beginning of each trial, a white cross appeared at the center of the screen. After a delay of a few seconds, which depends on the specific condition, a target (a colored disk of 0.7 cm radius) appeared at 10 cm from the center either upward or leftward, and signaled the start of the movement. Subjects were instructed to move the 1 cm-cross-shaped white cursor to the target within 1.5 s. Feedback was presented in two forms. First, the cursor was displayed early in the movement, while inside an invisible disk of 3.3 cm-radius centered at the home position (defined as the initial position of the cursor at the onset of target). Second, the cursor re-appeared 1.5 s after the start of movement for 0.5 s at the end-point of movement. To encourage faster reaction after appearance of the target, the cursor color became yellow if the subjects did not move within 1.0 s. If the subject did not move within 1.5 sec, the trial was considered a missed trial. After feedback presentation, a white circle with diameter proportional to the distance between the cursor and the home position helped the subjects to move back to the home position.

The targets appeared at a random position (uniform distribution) either upward (CCW rotation, green in Fig 1*C*), along an arc ranging from 60° to 120°, or leftward (CW rotation, blue in Fig 1*C*), along an arc ranging from 150° to 210°. Thus, after complete adaptation, the hand position required to hit the targets (goal positions in Fig 1*C*) for the two tasks were in two opposite ranges centered on 45° and 235°, respectively. Separation of the workspace (i.e., range of target and goal positions, see Fig 1*C*) between the tasks allowed all the subjects to succeed in dual adaptation [16, 23]. Variability in the target position at each trial was designed to make it more difficult for subjects to use a cognitive planning strategy that can occur with a fixed target position [24, 25]. In baseline and retention trials, target color was always red and appeared randomly along the leftward or the upward arcs.

### Data analysis

At each trial, we measured the directional error between the target and the final cursor position, which was calculated as the angle between the line connecting the center to the target position and the line connecting the center to the final cursor position. Data analysis, as well as model selection and parameter estimation (see below), were conducted based on the median of the bias-corrected movements from 15 subjects for each trial of each experiment group. The advantage of the median over the mean is to reduce the effect of outliers, composed of large overshoots or undershoots. We estimated the directional bias for each subject by taking the mean of the movement directions in the upward or leftward baseline trials, depending on the task, and subtracted this bias from the actual movement directions.

To compute initial learning rates, we fitted an exponential function to the directional errors of initial 10 trials of one task in the training block for each condition. Then, we estimated the initial learning rate as a slope of the tangential line of the fitted function at the first trial (in degrees per trial).

To estimate retention, we calculated the median of 5 trials in each retention test for each subject. Difference in overall performance in the 10 minute retention test was tested with one-way ANOVA. We then compared short-term forgetting, defined as the difference in performance in the immediate retention test and the 2-minute-post-training test for the different training conditions. We included random intercepts to account for inter-subject variability in performance (random intercept, *P* < 0.0001). We used the smallest BIC for mixed model selection and the Restricted Maximum Likelihood method with SPSS 18. Our criterion for significance was *P* < 0.05.

The effects of time and/or interference on between-trial forgetting during training were calculated by taking the differences in performance between experimental groups that differ in ITIs (SHORT-ITI vs. LONG-ITI, effect of time), or between experimental groups that differ in the presence of a second task (LONG-ITI vs. ALT, effect of interference), or both (SHORT-ITI vs. ALT, both). Total between-trial forgetting was then assessed by the area under the curve. To perform these subtractions, we used a bootstrap analysis: For each experimental condition, we generated 10,000 bootstrapped data sets. Each set consisted of random selection of the 15 subjects in each condition with replacement.

### Computational model

We previously proposed a multiple adaptation model with a parallel structure of fast and slow motor memories [21]. The model contains one common fast-updating fast-decaying process and multiple separate slow-updating slow-decaying processes. Here, we extended the previous model with multiple fast processes. The motor output at each trial *n* is given by:

$$y(n)=x_{\text{f}}{\left(n\right)}^{T}c_{\text{f}}(n)+x_{\text{s}}{\left(n\right)}^{T}c_{\text{s}}(n)$$ (1)

where **x**_f_ is a vector for multiple fast learning processes and **x**_s_ is a vector for multiple slow learning processes. In the case of two tasks, there are two fast and slow processes, **x**_f_ = (*x*_f1_ *x*_f2_)^*T*^, **x**_s_ = (*x*_s1_ *x*_s2_)^*T*^. In the original model, the contextual cue only with the slow processes, **c**_s_ addressed either of processes such that **c**_s_ = (1 0)^*T*^ for task 1 and c_s_ = (1 0)^*T*^ for task 2, where we assumed perfect switching, i.e., no interference or transfer between the slow processes. Additionally, the original model assumed full interference in the fast process. However, in the extended model, we also generalized the degree of interference both in the fast and in the slow processes. The contextual cue vectors of Eq 1 are defined as **c**_f_ = (1 *q*_f_)^*T*^ or **c**_f_ = (*q*_f_ 1)^*T*^ and **c**_s_ = (1 *q*_s_)^*T*^ or **c**_s_ = (*q*_s_ 1)^*T*^, where the free parameter *q*_f_ and *q*_s_ modulate the degree of interference, and range from 0 (no interference) to 1 (full interference). Note that in our experimental design with opposite rotations, -45° and +45°, positive values of *q*_f_ and *q*_s_ represent interference because the sign of the state of the fast and slow processes for task 2, *x*_f2_ and *x*_s2_, are opposite to those of task 1, *x*_f1_ and *x*_s1_.

To estimate the amount of memory decay as a function of time independently of trials, we updated the model by replacing the forgetting rate parameters with exponential decay terms [10, 26]. The update equations from trial *n* to trial *n*+1 for the fast and the slow processes are then as follows:

$$x_{\text{f}}(n+1)=x_{\text{f}}(n)e^{-T(n)/\tau_{\text{f}}}+\beta_{\text{f}}\cdot e(n)\cdot c_{\text{f}}(n)$$ (2)

$$x_{\text{s}}(n+1)=x_{\text{s}}(n)e^{-T(n)/\tau_{\text{s}}}+\beta_{\text{s}}\cdot e(n)\cdot c_{\text{s}}(n)$$ (3)

$$\text{c}.\text{t}.\;\tau_{\text{f}}<\tau_{\text{s}},\;\beta_{\text{f}}>\beta_{\text{s}}$$ (4)

where *T*(*n*) is the inter-trial interval following trial *n* (in seconds); *β*_f_, *τ*_f_, *β*_s_ and *τ*_s_ are four free parameters with the constraints c.t., which are learning rates and time constants for the fast and slow learning processes, respectively. The motor error, *e* is the difference between the external perturbation, *f* and the motor output, *y*. We assumed no difference in task difficulty between the two tasks, CCW and CW in ALT. To test this assumption, we calculated the initial learning rate from the first 15 trials and as well as final performance as the median of the last 15 trials in the two tasks. We found no significant difference between the two tasks in the initial learning rate (*P* = 0.093) and the final performance (*P* = 0.308). Additionally, we found no significant difference in performance between the two tasks during training trials (paired two tailed *t*-test, *P* = 0.216). We thus used the same learning rates (*β*_f_, *β*_s_) and time constants (*τ*_f_, *τ*_s_) for the two tasks. Note that in one-task conditions, such as SHORT-ITI or LONG-ITI, the model reduces to the 1-fast-1-slow model of [12] with the exponential decay terms.

### Model comparison and parameter fitting

We conducted a model comparison analyses with six models, which are summarized in Table 1. We fitted the listed free parameters of each model to data from the three experimental conditions. Note that model 6 is the generalized model described as in Eqs 1–4 and all the other models are variants of the model with fewer free parameters. Also, note all models can account for adaptation to two perturbations, but that two data sets (SHORT-ITI and LONG-ITIT) contain data only for one task. We therefore only estimated the interference parameters with the ALT dataset. We did not include models without interference parameters (*q*_f_ = *q*_s_ = 0), because we found a significant effect of interference between the two tasks during training (see Results). For model parameter estimation, we used the MATLAB *fmincon* function, which minimizes the root mean squared error (RMSE) between the observed median data *y*(*n*), and the model prediction $\hat{y}(n)$, at trial *n*. Model comparison was based on the Bayesian Information Criterion (BIC) as follows.

$$\text{BIC = }N\text{ln}\left[\frac{1}{N}\sum_{n=1}^{N}{\left(y(n)-\hat{y}(n)\right)}^{2}\right]+k\text{ln}N$$ (5)

where *k* is the number of parameters in the model, and *N* is the number of trials used for model fitting. The first term of the BIC is the fitting error (in brackets), which decreases with more free parameters. However, a complex model with too many parameters can lead to over-fitting and little generalization. By penalizing the number of free parameters in the second term, the BIC allows us to find parsimonious model, with a good balance between fitting errors and model complexity.

**Table 1:** Description of tested models. Each of candidate models was fitted to all the data from the three groups (*N* = 300). For the groups with one task, SHORT-ITI and LONG-ITI, the interference parameters were ignored in models.

Name	Description	Parameters
	Two interfering slow processes	
Model 1	*y*(*n*) = **x**_s_(*n*)^*T*^ **c**_s_(*n*)	*β*_s_, *τ*_s_, *q*_s_
	**c**_s_(*n*) = (1 *q*_s_)^*T*^ **c**_s_(*n*) = (*q*_s_ 1)^*T*^	*k* = 3
	Common fast process and two independent slow processes	
Model 2	*y*(*n*) = *x*_f_(*n*)+**x**_s_(*n*)^*T*^ **c**_s_(*n*)	*β*_f_, *τ*_f_, *β*_s_, *τ*_s_
	**c**_s_(*n*) = (1 0)^*T*^ **c**_s_(*n*) (0 1)^*T*^	*k* = 4
	Common fast process and two interfering slow processes	
Model 3	*y*(*n*) = *x*_f_(*n*)^*T*^+**x**_s_(*n*)^*T*^ **c**_s_(*n*)	*β*_f_, *τ*_f_, *β*_s_, *τ*_s_, *q*_s_
	**c**_s_(*n*) = (1 *q*_s_)^*T*^ **c**_s_(*n*) = (*q*_s_ 1)^*T*^	*k* = 5
	Two interfering fast processes and two independent slow processes	
Model 4	*y*(*n*) = **x**_f_(*n*)^*T*^ **c**_f_(*n*)+**x**_s_(*n*)^*T*^ **c**_s_(*n*)	*β*_f_, *τ*_f_, *β*_s_, *τ*_s_, *q*_f_
	**c**_f_(*n*) = (1 *q*_f_)^*T*^ **c**_f_(*n*) = (*q*_f_ 1)^*T*^ **c**_s_(*n*) = (1 0)^*T*^ **c**_s_(*n*) (0 1)^*T*^	*k* = 5
	Two independent fast processes and two interfering slow processes	
Model 5	*y*(*n*) = **x**_f_(*n*)^*T*^+**c**_f_(*n*)+**x**_s_(*n*)^*T*^ **c**_s_(*n*)	*β*_f_, *τ*_f_, *β*_s_, *τ*_s_, *q*_s_
	**c**_f_(*n*) = (1 0)^*T*^ **c**_f_(*n*) = (0 1)^*T*^ **c**_s_(*n*) = (1 *q*_s_)^*T*^ **c**_s_(*n*) = (*q*_s_ 1)^*T*^	*k* = 5
	Two interfering fast processes and two interfering slow processes	
Model 6	*y*(*n*) = **x**_f_(*n*)^*T*^ **c**_f_(*n*)+**x**_s_(*n*)^*T*^ **c**_s_(*n*)	*β*_f_, *τ*_f_, *β*_s_, *τ*_s_, *q*_f_, *q*_s_
	**c**_f_(*n*) = (1 *q*_f_)^*T*^ **c**_f_(*n*) = (*q*_f_ 1)^*T*^ **c**_s_(*n*) = (1 *q*_s_)^*T*^ **c**_s_(*n*) = (*q*_s_ 1)^*T*^	*k* = 6

For each experimental condition, we generated 10,000 bootstrapped data sets: Each set consisted of random selection out of the 15 subjects in each condition with replacement. We then fitted the candidate models to the median of the selected (bootstrapped) data. We then calculated the BIC of the bootstrapped data set for the candidate models (see Table 1) and the conditions (i.e., SHORT-ITI, LONG-ITI, and ALT). We selected the model with the lowest BICs by performing paired bootstrap *t*-tests [27] (see details in [21]) and reported bootstrap *P-*value as the proportion of the bootstrapped data preferring (i.e., with lower BIC) the compared model versus the selected model. To select the model(s) with the significantly lowest BIC, we performed a pairwise bootstrap *t*-test for every pair of models, taking a *P*-value at threshold *P* < 0.001 to account for multiple comparisons.

## Results

**Fig 2:**
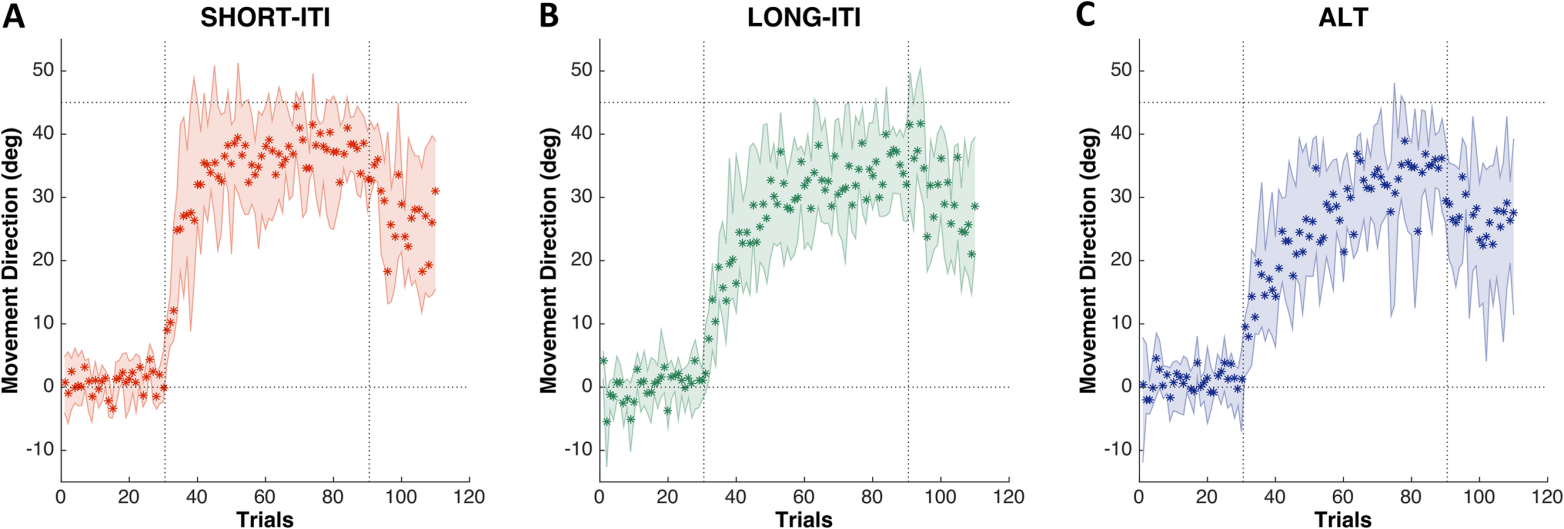
Observed subject behaviors in three experimental conditions. SHORT-ITI (***A***), LONG-ITI (***B***), and ALT (***C***). Star symbols: median subject performance. Shaded area: inter-quartile (25–75%) ranges.

Fig 2 shows actual adaptation (medians with inter-quartile ranges) of three experimental groups. Adaptation for task 2 in ALT was symmetric to task 1 across entire trials and subjects, showing no significant difference between the two tasks, as we assumed the same learning and forgetting rates for the two tasks (see Methods).

### Initial learning rate analysis

**Fig 3:**
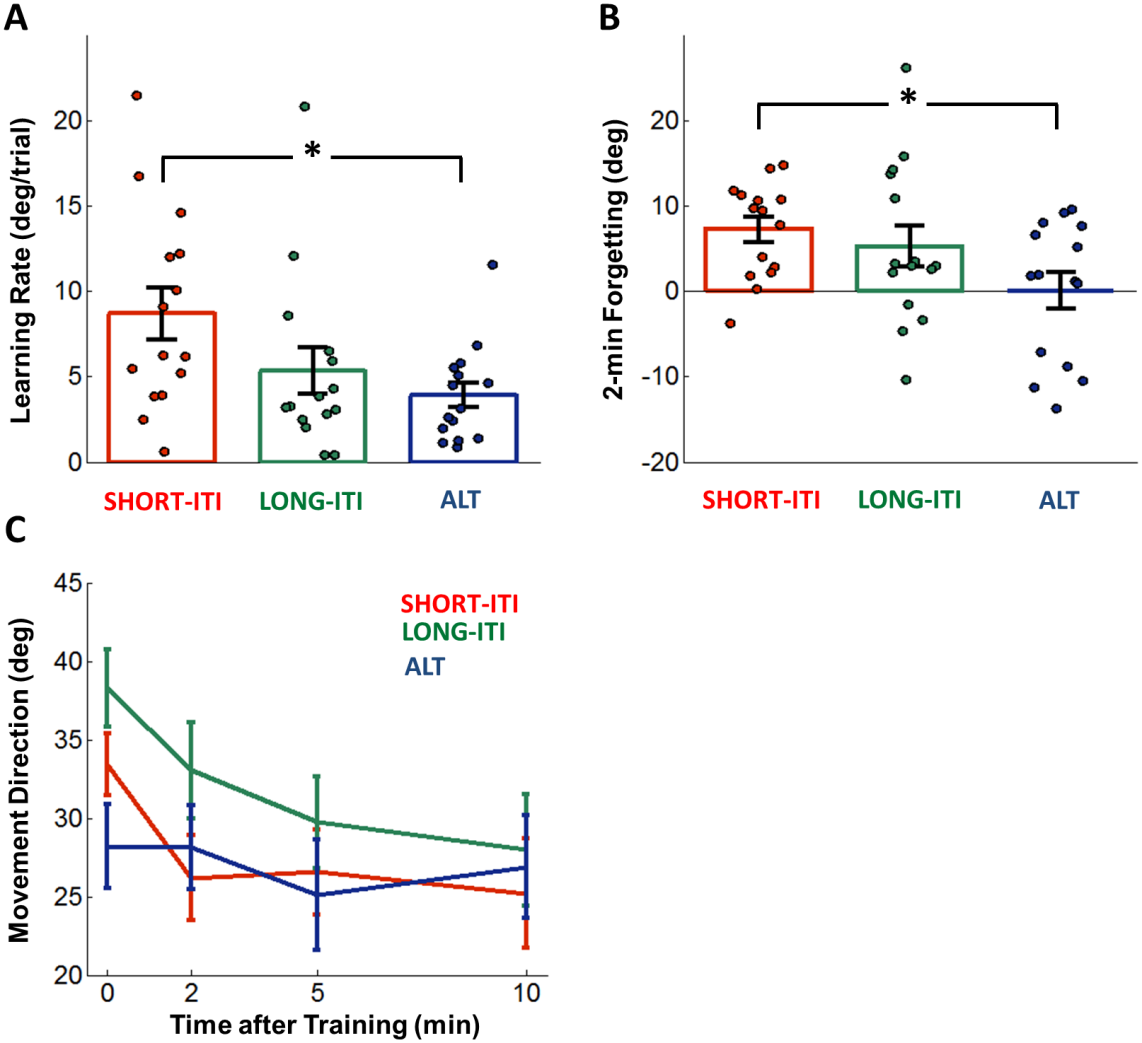
Comparison of initial learning and retention. ***A***: Initial learning rates (performance increase per trial) obtained from the initial 15 trials in the training block. ***B***: Forgetting in the two minute post-training. Forgetting was measured as a difference between zero and two minute retention tests post-training (see Methods). ***C***: Overall retention in 0, 2, 5, and 10 minute post-training. In Fig 4*A* and 4*B*, circles indicate performance of individual subjects.

As can be observed in Fig 2, subjects adapted at different rates in the different conditions. A Kruskal-Wallis test confirmed that initial learning rates differed across experiment groups (mean ± SEM; SHORT-ITI: 8.69 ± 1.50°/trial, LONG-ITI: 5.34 ± 1.36°/trial, ALT: 3.91 ± 0.74°/trial, *P* = 0.027, Fig 3*A*). A post-hoc test showed that the initial learning rate of SHORT-ITI was larger than that of ALT (rank-sum test, *P* = 0.038, Bonferroni-corrected). Although the learning rate of LONG-ITI was not significantly different from learning rates in the other two conditions (SHORT-ITI/LONG-ITI, *P* = 0.13 and ALT/LONG-ITI, *P* = 0.92), the mean value was between those of the other two groups. Despite this initial differences in learning rates, there was no difference in final performance (median over the last five training trial) across groups (mean ± SEM; SHORT-ITI: 37.4 ± 2.18°, LONG-ITI: 35.4 ± 1.70°; ALT: 32.9 ± 2.24°, Kruskal-Wallis test, *P* = 0.45).

### Retention analysis

Mixed model analysis for retention data between the first two tests (at 0 and 2 minutes) showed that training condition (*P* = 0.037), time of testing after training (*P* = 0.001), and the interaction of condition and time of testing (*P* = 0.046) contributed to forgetting significantly. The short-term forgetting within 2 minutes was significantly different from 0 in SHORT-ITI (7.21 ± 1.44°, *P* = 0.001) and LONG-ITI (5.24 ± 2.42°, *P* = 0.013). Importantly, the forgetting was not different from 0 in ALT (0.057 ± 2.11°, *P* = 0.97) (Fig 3*B* and 3*C*). The forgetting was larger in SHORT-ITI compared to ALT (*P* = 0.017), and with a trend towards being greater in LONG-ITI than ALT (*P* = 0.080), but was not different between SHORT-ITI and LONG-ITI (*P* = 0.50) (Fig 3*B*).

There was an effect of condition on overall performance in the 0, 2, 5, and 10 minute post-tests (one way with condition as factor ANOVA, *P* < 10^−4^) (Fig 3*C*). Overall retention performance in LONG-ITI was 31.62 ± 0.82°, and was greater than in SHORT-ITI at 28.32 ± 0.87° (*P* = 0.02, Bonferroni-corrected) as predicted. However, unlike our predictions, overall retention performance in LONG-ITI was greater than in ALT (26.18 ± 0.89, *P* < 0.001), and there was no difference between SHORT-ITI and ALT (*P* > 0.05). This decrease of overall performance in ALT was not due to time-based forgetting, because it was already present in the immediate retention test: Performance in the immediate retention test at 0 minute was smaller in ALT than in LONG-ITI (Fig 3*C*, *P* = 0.01). This lower performance in ALT in the immediate test was due to a (marginally significant) performance drop of performance in ALT from the last 5 trials at the end of training (at which time there was no difference (two tailed *t*-test, *P =* 0.40) between ALT and LONG-ITI) to the first immediate test (*P* = 0.05)

### Dissociation of time and interference-induced between-trial forgetting

**Fig 4:**
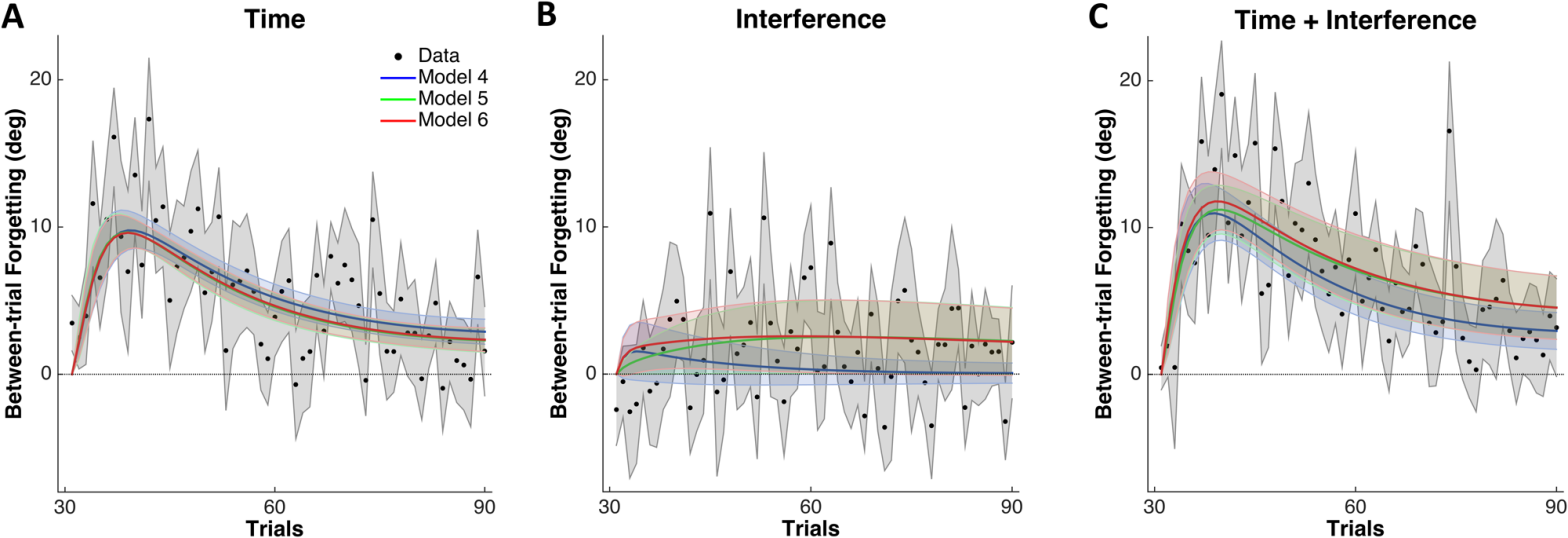
Estimated effects of time and/or interference on between-trial forgetting with data and predictions from the three selected models. ***A***: Time effect as the difference in training performance between SHORT-ITI and LONG-ITI. ***B***: Interference effect as the difference in training performance between and LONG-ITI and ALT. ***C***: Combined effect of time and interference as the difference in training performance between SHORT-ITI and ALT. Black: data, blue: model 4 (two interfering fast processes and two independent slow processes), green: model 5 (two independent fast processes and two interfering slow processes), red: model 6 (two interfering fast processes and two interfering slow processes). Dots and lines indicate the means, shaded areas indicate the ± S.D. from 10,000 bootstrapped samples.

Between-trial forgetting due to either time or interferences or both during training was assessed by taking the difference in performance between conditions (see Methods). The total amount of between-trial forgetting due to time (Fig 4*A*) or interference (Fig 4*B*) or both (Fig 4*C*) was significantly larger than zero (areas under the curve, two tailed *t*-test *P* < 10^−14^ for forgetting due to time and *P* = 0.013 for forgetting due to interference, and *P* < 10^−14^ for forgetting due to both time and interference. Note how, on all the panels of Fig 4, between-trial forgetting initially increased but then converged to zero toward the end of training, showing that the between-trial forgetting occurs at relatively fast time scale during training.

### Model comparison analysis

Table 2 summarizes the results of the model comparison analysis from 10,000 bootstrapped data for the six models, with BICs. The BICs for three models (models 4, 5, and 6, all with some degree of interferences in the fast, slow, or both processes) were significantly lower (*P* < 0.05) than BICs of model 1 (Two interfering slow processes), of model 2 (Common fast and two independent slow processes), and of model 3 (Common fast and two interfering slow processes)—BIC of model 4 was marginally lower than those of model 3 (*P* = 0.081). However, BICs were not significantly different among the best fitting models 4, 5, and 6. Specifically, BIC of the model with the lowest mean BIC (model 5, Two independent fast processes and two interfering slow processes) was not significantly different (*P* = 0.258) from that of model 4 (Two interfering fast processes and two independent slow processes), and (*P* = 0.090) that of model 6 (Two interfering fast processes and two interfering slow processes). Because these three models differ in interference parameters, we compared how they predict between-trial forgetting. Between-trial forgetting due to interference predicted by model 4 (Two interfering fast processes and two independent slow processes) was slightly less than that of models 5 (Two independent fast processes and two interfering slow processes) and model 6 (Two interfering fast processes and two interfering slow processes). In addition, between-trial forgetting in model 4 converged to zero towards the end of training because this model assumed no interference between the slow processes. Note however that, as shown by our model comparison analysis (see above), there was no significant difference among the three models. Thus, although we can conclude that interferences are needed to account for our data, it is unclear from these results whether interferences occur in fast, slow, or both processes.

**Table 2:** Results of the model comparison analysis. For the tested models described in Table 1, BICs and the confidence intervals of the estimated parameters from 10,000 bootstrapped data sets were presented. BIC of models 4, 5, 6 with interference parameter(s) was significantly lower than that of the other models.

Name	BIC	*τ*_f_(s)	*β*_f_	*q*_f_	*τ*_s_(s)	*β*_s_	*q*_s_
**Model 1**	1105.4			0	660.6–1300	0.062–0.116	0–0.529
**Model 2**	1056.4	36.8–95.1	0.072–0.183	1	893.8–2020	0.044–0.076	0
**Model 3**	1055.0	36.4–73.3	0.074–0.182	1	892.6–1990	0.047–0.084	0–0.422
**Model 4**	1009.7	26.3–88.8	0.12–0.343	0–0.556	1384–7367	0.030–0.057	0
**Model 5**	988.8	23.2–83.2	0.12–0.377	0	1468–10013	0.034–0.064	0–0.551
**Model 6**	992.9	26.3–84.8	0.12–0.351	0–0.449	1470–10158	0.034–0.064	0–0.548

Table 2 also shows parameter estimates with 95% confidence intervals for all models. Note that estimated time constant of the fast process is roughly between 0.5 and 1.5 minute for all models. Time constant for the slow process is roughly above 20 minutes. Finally, the estimated interference parameters in the fast process are lower in models 4 to 6 than in models 2 and 3 with full interference: While the full interference models 2 and 3 assume *q*_f_ = 1, the estimated interference parameters was between 0–0.556 for model 4 (interference in fast process only) and between 0–0.449 for model 6 (interference in both processes).

## Discussion

We studied and compared the effect of temporally spaced presentations and the effect of intercalating a second task during visuomotor adaptation with three conditions SHORT-ITI, LONG-ITI, and ALT. The only difference between SHORT-ITI and LONG-ITI was the inter-trial interval (ITI), and the only difference between LONG-ITI and ALT was the intercalation of the second task. Thus, this experimental design allowed us to dissociate the effects of forgetting induced by time and by interference.

### Discussion of experimental results

Performance changes during training were as predicted by the BTF-LTR hypothesis: Initial adaptation rate was largest in SHORT-ITI, smallest in ALT, and intermediate in LONG-ITI. Similarly, short-term forgetting in the retention tests (i.e., during the first 2 minutes) was as predicted by the BTF-LTR hypothesis, with the notable finding that there was no forgetting in the retention tests in the ALT condition. Overall, short-term forgetting was largest in SHORT-ITI, and smallest in ALT, and intermediate in LONG-ITI. In addition, our design allowed us to estimate the effect of between-trial forgetting due to time by comparing training performance in SHORT-ITI to LONG-ITI (Fig 4*A*) and due to interference by comparing training performance in LONG-ITI to ALT (Fig 4*B*). As predicted by the BTF-LTR hypothesis, between-trial forgetting was significantly greater than zero for both time and interference, although the effect of time appears greater than that of interference.

However, overall retention performance in all retention tests was best in LONG-ITI than in the other two conditions while there was no difference between ALT and SHORT-ITI. This result contradicts, at first sight, prediction from the BTF-LTR and results from the literature on the contextual interference effect of greater retention in condition with alternating tasks [4, 28]. In our experiment, it is notably possible that training was too short to observe a strong contextual interference effect. In addition, this may be due to differences in testing conditions between our study and previous studies: In contextual interference effect studies, there are typically four groups of subjects, two per training condition (alternating and non-alternating): one group with testing schedule identical to training schedule, and the other group with testing condition in the other training schedule [28]. Because we had three different conditions, such an exhaustive testing was not practical and would have led to multiple comparisons. We thus chose to give tests with a single schedule: the tests had the same ITI as in ALT, but with a single task. Thus, although ITI in ALT retention test was identical to ITI in training, retention in ALT may have been affected by the change of context in the tests: although both tasks were practiced during training, a single task was tested following ALT training (in order to keep tests identical across conditions). Such “encoding specificity” is known to affect retrieval of episodic memory [29]. A similar phenomenon may be at play here, partially accounting for the marginal performance drop from the end of training to the immediate retention. However, our results showed that once this change has been recognized, the remaining decay was slow. A recent study showed how the context where learning occurred more strongly affects update and decay of the motor memory associated with the context [30]. Although results from this study may appear to contradict our results, there are important differences. In this previous study, the context referred to movement directions, and not the number of tasks during training as in our study. In addition, the drop in performance that we observed following training in ALT was almost instantaneous (ITI between last training trial and first retention trial was only 5.2 second) and not due to a slower decay process as in the previous study.

### Interpretation of experimental results in light of the model comparison

Model 1, the model with the worst fit (i.e. highest BICs), is the only model that contains only slow processes. All better fitting models contain fast and slow processes. This points for at least two time scales in motor adaptation, a well-known result [31–34], which accounts for the fast initial adaptation followed by slow and gradual adaptation observed in Fig 2.

The two next best fit models contain a single fast process, thus assuming full interference in the fast process, and two independent slow (model 2) or interfering slow (model 3) processes. These models, which laid the ground for the BTF-LTR hypothesis (reduced adaptation due to both spacing and interferences lead to greater retention), were tested because they extend the models that we developed in previous works [21, 23]. The BICs for these models are however higher than models with non-complete interference in the fast process (models 4, 5, and 6). These models support the BTF-LTR and our results because the mean estimated time constants for the fast and slow processes in models 2 to 6 are approximately 1 minute for *τ*_f_ and 20 minutes for *τ*_s_, respectively, presumably implying that the fast process almost entirely decay in the first 2 minutes. Thus, performance at the time of the 2 minute retention tests is mostly due to the slow process in all conditions. Because of time-based between-trial forgetting in the LONG-ITI, performance during training is lower, allowing for greater errors to update the slow process, and thus better long-term retention than in the SHORT-ITI. Similarly, because of both time-based between-trial forgetting and additional interference based forgetting in the ALT, performance during training is even lower, allowing for even greater errors to update the slow process and very little forgetting in retention tests.

However, our results show that the degree of interferences in these models is not as high as we previously envisioned: As by parameter estimation, the interference parameter in the fast process is estimated to be between 0–0.556 (model 4) and between 0–0.449 (model 6), whereas models 2 and 3 assumed it was equal to 1. Such relatively small effect of interference in the models is seen in Fig 4*B*. The degree of interference is dependent on the ITI, with greater interference for small it is between conflicting tasks, but also on the task set up. In our design, we separated the workspace to allow all subjects to learn both tasks, as we noticed in piloting that with overlapping workspaces, a number of subjects were unable to learn both tasks (for similar results, refer to [19]). In addition, the three models with best fits, models 4, 5, and 6, had either interference in the fast, slow, or both processes, respectively. Because there were not significant differences among the BICs of these models, the present results do not allow us to conclude in which process most (all) of the interferences occur. Nevertheless, our results in the ALT condition, as well as previous results showing adaptation to two tasks concurrently (e.g. [15–17]), show that the slow process are protected from interference to the extent that dual adaptation is possible, thus pointing to a main locus of interferences in the fast process. This is consistent with previous studies showing that the fast process is linked to working memory, which is vulnerable to interference. In particular, the early phase of adaptation has been shown to correlate with tests of visuo-spatial working memory [22, 34], and the fast process can be interfered by a task that engages declarative memory [35]. In contrast, there are data showing that multiple slow processes are protected from interference [21, 36]. In any case, future work is needed to better understand in which experimental conditions, and at which time scale interferences occur during visuomotor adaptation.

### Comparison with previous works

Several recent studies explored the effect of task schedules in motor learning or adaptation, and are related to the present study. Two studies reported no significant difference in learning rates during practice when the time between trials was greater than 1 second [37, 38]. Similarly, we found no difference in the initial learning rates between SHORT-ITI and LONG-ITI. Unlike our results, Huang et al. (2007) showed increased learning rate during practice with a longer ITI group compared to a shorter ITI group [39], which cannot be explained by multi-rate learning models with fixed learning rates. However, they calculated learning performance by averaging 32 trials, which might not capture dynamic change of the fast process with a time constant of less than a minute. Kording et al. (2007) proposed that the spacing effect is due to increase in learning rates of the slower processes during spaced adaptation [13]. Although this is one possible explanation, 1-fast-1-slow models do not require modifiable learning rates to explain the spacing effect: longer ITI induces more forgetting in the fast process, which in turn results in larger errors, and thus greater update in the slow process. In our previous study [22], we compared the contextual interference effect in learning to generate three specific force profiles in healthy individuals and individuals with chronic stroke. We showed that individuals with chronic stroke, who had low visuo-spatial working memory, exhibited little long-term forgetting after either blocked or random schedules. This finding was predicted based on simulations of the Lee and Schweighofer model [21] with decrease in fast process update.

## Conclusions

In sum, our results strongly support the BTF-LTR hypothesis: the spacing and the contextual interference effects are largely based on forgetting between presentations of the same task during training. However, the specific mechanisms underlying the enhancement of long-term memory differ in the “forgetting-reconstruction” hypothesis [8] and in the BTF-LTR hypothesis. According to the forgetting-reconstruction hypothesis, forgetting in working memory between spaced presentations necessitates retrieval from long-term memory, which increases long-term retention. In the state-space models and the BTF-LTR hypothesis, forgetting between spaced presentations leads to greater errors during training, which increases the update of the slow process, and in turn leads to decreased forgetting. Note that our account of the contextual interference effect does not exclude additional explanations, such as the “elaboration-distinctiveness” or “deficient processing” [7, 40]. Further studies are needed to dissociate the possible roles of additional mechanisms in both the spacing and the contextual interference effects.
